# Both the concentration and redox state of glutathione and ascorbate influence the sensitivity of arabidopsis to cadmium

**DOI:** 10.1093/aob/mcv075

**Published:** 2015-06-12

**Authors:** Marijke Jozefczak, Sacha Bohler, Henk Schat, Nele Horemans, Yves Guisez, Tony Remans, Jaco Vangronsveld, Ann Cuypers

**Affiliations:** ^1^Hasselt University, Centre for Environmental Sciences, Agoralaan Building D, B-3590 Diepenbeek, Belgium,; ^2^Free University of Amsterdam, Institute of Molecular and Cellular Biology, De Boelelaan 1085, NL-1081 HV Amsterdam, The Netherlands,; ^3^Belgian Nuclear Research Centre, Biosphere Impact Studies, Boeretang 200, B-2400 Mol, Belgium and; ^4^University of Antwerp, Department of Biology, Middelheim campus, G.U616, Groenenborgerlaan 171, B-2020 Antwerp, Belgium

**Keywords:** Antioxidative defence, *Arabidopsis thaliana*, ascorbate, cadmium stress, cellular redox state, chelation, glutathione, phytochelatins, reactive oxygen species

## Abstract

**Background and Aims** Cadmium (Cd) is a non-essential trace element that elicits oxidative stress. Plants respond to Cd toxicity via increasing their Cd-chelating and antioxidative capacities. They predominantly chelate Cd via glutathione (GSH) and phytochelatins (PCs), while antioxidative defence is mainly based on the use and recycling of both GSH and ascorbate (AsA), complemented by superoxide dismutase (SOD) and catalase (CAT). In addition, both metabolites act as a substrate for the regeneration of other essential antioxidants, which neutralize and regulate reactive oxygen species (ROS). Together, these functions influence the concentration and cellular redox state of GSH and AsA. In this study, these two parameters were examined in plants of *Arabidopsis thaliana* exposed to sub-lethal Cd concentrations.

**Methods** Wild-type plants and mutant arabidopsis plants containing 30–45 % of wild-type levels of GSH (*cad2-1*) or 40–50 % of AsA (*vtc1-1*), together with the double-mutant (*cad2-1 vtc1-1*) were cultivated in a hydroponic system and exposed to sub-lethal Cd concentrations. Cadmium detoxification was investigated at different levels including gene expression and metabolite concentrations.

**Key Results** In comparison with wild-type plants, elevated basal thiol levels and enhanced PC synthesis upon exposure to Cd efficiently compensated AsA deficiency in *vtc1-1* plants and contributed to decreased sensitivity towards Cd. Glutathione-deficient (*cad2-1* and *cad2-1 vtc1-1*) mutants, however, showed a more oxidized GSH redox state, resulting in initial oxidative stress and a higher sensitivity to Cd. In order to cope with the Cd stress to which they were exposed, GSH-deficient mutants activated multiple alternative pathways.

**Conclusions** Our observations indicate that GSH and AsA deficiency differentially alter plant GSH homeostasis, resulting in opposite Cd sensitivities relative to wild-type plants. Upon Cd exposure, GSH-deficient mutants were hampered in chelation. They experienced phenotypic disturbances and even more oxidative stress, and therefore activated multiple alternative pathways such as SOD, CAT and ascorbate peroxidase, indicating a higher Cd sensitivity. Ascorbate deficiency, however, was associated with enhanced PC synthesis in comparison with wild-type plants after Cd exposure, which contributed to decreased sensitivity towards Cd.

## INTRODUCTION

Cadmium (Cd) pollution is a worldwide environmental and health concern. Even low concentrations of Cd in the environment are associated with increased mortality (Nawrot *et al*., 2008; Maruzeni *et al*., 2014). Mainly areas with high industrial or agricultural activities show elevated Cd levels in the soil, disturbing the entire ecosystem (Vangronsveld and Clijsters, 1994; Chary *et al*., 2008). At the cellular level, Cd toxicity elicits oxidative stress in organisms (Bertin and Averbeck, 2006; Cuypers *et al*., 2009). In plants, recent studies have focused on the Cd-induced oxidative challenge and the role of reactive oxygen species (ROS) in signalling processes, which control cellular and molecular responses in plants (Keunen *et al*., 2013; Smeets *et al*., 2013). Since ROS are continuously produced during normal cell metabolism, their basal levels are rigorously restrained. They are key players in regulating plant development and responses to environmental changes, including abiotic stress (Mittler *et al*., 2011; Suzuki *et al*., 2012). During metal stress, accumulation of ROS could evoke severe oxidative damage or important oxidative signalling to activate defence mechanisms. Whether ROS will act as damaging or signalling factors depends on the balance between ROS production and scavenging (Gratao *et al*., 2005; Miller *et al*., 2008; Keunen *et al*., 2011*a*).

Gadjev *et al.* (2006) identified five gene transcripts that were upregulated >5-fold upon several conditions resulting in oxidative stress: a gene upregulated by oxidative stress (*UPOX*, *AT2G21640*), a defensin-like gene (*defensin-like*, *AT2G43510*), two genes with unknown function (*AT1G19020* and *AT1G05340*) and a gene of the Toll-Interleukin-1 class (*TIR*, *AT1G57630*). These genes are considered hallmark genes for the general oxidative stress response (Mittler *et al*., 2004; Gadjev *et al*., 2006). In the case of Cd exposure, the plant’s major cellular defences are to prevent accumulation of free metal ions and to neutralize excessive ROS.

Glutathione (GSH) is the most ubiquitous and abundant non-protein thiol in plant cells, containing a free thiol group on cysteine. Cadmium has a high affinity for the thiol group of GSH and phytochelatin synthase (PCS). Binding results in the activation of PCS, which polymerizes GSH molecules into phytochelatins (PCs). The latter is a fast and efficient defence against Cd toxicity. Thiols are also able to undergo redox reactions, providing GSH with both chelating and antioxidant properties (Brunetti *et al*., 2011; Jozefczak *et al*., 2012; Zagorchev *et al*., 2013; Jozefczak *et al*., 2014).

In plants, antioxidative responses are largely based on the use and recycling of both GSH and ascorbate (AsA), complemented by superoxide dismutase (SOD) and catalase (CAT). The SODs constitute the first line of defence against superoxide (O_2_·^–^), the first ROS intermediate. These enzymes are classified into three groups based on their metal co-factor: iron (FeSOD), copper/zinc (CuZnSOD) and manganese SOD (MnSOD). These redox-active metals dismutate O_2_·^–^ and produce the relatively stable hydrogen peroxide (H_2_O_2_). Both CAT and ascorbate peroxidase (APx) are major H_2_O_2_ scavengers preventing the formation of toxic hydroxyl radicals (·OH) (Mittler *et al*., 2004; Halliwell, 2006). In contrast to APx, CAT has a low affinity for H_2_O_2_ but a high reaction speed and no limitation by its substrate. These different characteristics make APx a good candidate for ROS fine tuning, while CAT might be responsible for removing the excess ROS (Cuypers *et al*., 2011). As stated before, GSH has primary antioxidant functions but it also acts as a substrate for the regeneration of other essential antioxidants such as glutaredoxins and AsA. In the AsA–GSH cycle, both metabolites are successively oxidized and reduced, allowing APx to neutralize H_2_O_2_. Oxidized forms of GSH [i.e. glutathione disulphide (GSSG)] and AsA [i.e. dehydroascorbate (DHA)] are recycled by NADPH-dependent glutathione reductase (GR) and GSH-dependent dehydroascorbate reductase (DHAR), respectively (Mittler *et al*., 2004; Jozefczak *et al*., 2012). Apart from its function as a substrate in the AsA–GSH cycle, AsA also directly neutralizes ROS non-enzymatically and it is involved in the regeneration of α-tocopherol, which scavenges both ROS and lipid peroxyl radicals, and zeaxanthin, which is involved in the photoprotective xanthophyll cycle (Bielen *et al*., 2013).

In order to maintain protein structure and function, a reducing intracellular environment is essential. Both GSH/GSSG and AsA/DHA are major cellular redox buffers. Changes in the ratios of the reduced and oxidized forms reflect cellular toxicity and have been associated with redox signalling (Foyer and Noctor, 2005; Jozefczak *et al*., 2012; Zagorchev *et al*., 2013). The exact mechanism of this signalling system is still under intense investigation. Therefore, in the present study, both the concentration and redox state of GSH and AsA were investigated in roots and leaves of Cd-exposed *Arabidopsis thaliana* plants and related to downstream molecular responses. To increase our knowledge concerning GSH- and AsA-related pathways, Cd-induced responses were compared between wild-type and mutant plants deficient in either one or both metabolites.

## MATERIALS AND METHODS

### Plant material, growth conditions and harvest

Wild-type (Lehle seeds, Round Rock, TX, USA) and mutant *A. thaliana* (ecotype Columbia) seeds were surface sterilized and grown in a hydroponic culture [Hoagland solution; 12 h/21 °C day, 12 h/17 °C night regime; 170 μmol m^–2 ^s^–1^ light at the leaf level delivered by cool white fluorescent lamps (L 140 W/20SA, Osram, Augsburg); and 65 % relative humidity; Smeets *et al.* (2008)]. The *cad2-1* mutant was provided by Dr Christopher Cobbett (Melbourne University, Australia). This mutant was selected in a screen for Cd sensitivity and carries a mutation in the GSH biosynthesis gene γ-glutamylcysteine synthetase (*GSH1*, *AT4G23100*), resulting in approx. 30 % of wild-type GSH levels (Cobbett *et al*., 1998). The *vtc1-1* mutant was obtained from the European Arabidopsis Stock Centre (uNASC ID: N8326, UK). This mutant was selected in a screen for vitamin C deficiency and carries a mutation in the AsA biosynthesis gene GDP-d-mannose pyrophosphorylase (*GMP*, *AT2G39770*), resulting in approx. 40–50 % of wild-type AsA levels (Conklin *et al*., 1999). The *cad2-1 vtc1-1* double mutant was provided by Dr Mark Aarts (Wageningen University, The Netherlands; Clerkx *et al.*, 2004). After a period of 3 weeks growth, plants were exposed to 0, 1 or 5 μm Cd supplied as CdSO_4_ in the Hoagland solution. After 72 h of exposure, the aerial part and roots were separated and dried prior to element determination or snap-frozen in liquid nitrogen, then stored at –80 °C, prior to the other analysis.

### Element determination

During harvest, roots were rinsed with distilled water and incubated for 30 min in an ice-cold desorbing solution of 5 mm PbNO_3_. Subsequently, roots were washed three times in distilled water and once in Millipore water. Leaves were rinsed three times with Millipore water. The fresh plant material was dried at 60 °C, weighed, and digested in 70–71 % HNO_3_ using a heat block. Element concentrations were measured via inductively coupled plasma-optical emission spectrometry (ICP-OES 710, Agilent Technologies, Australia).

### Metabolite measurements

Analyses of AsA, GSH and PC contents were performed with reverse-phase HPLC as previously described (Semane *et al*., 2007). For total AsA and GSH measurements, frozen tissues were ground in liquid nitrogen and homogenized in 6 % (w/v) meta-phosphoric acid. Samples were centrifuged (15 min, 14 000 *g*, 4 °C) and the supernatant was incubated with dithiothreitol. After 15 min, this reduction was stopped by adding acetonitrile and the samples were separated at 40 °C on a reverse-phase C_18_ column (type Polaris 3 C_18_, 3 μm, 100 × 4·6 mm, Varian, The Netherlands) with an isocratic flow of 0·8 mL min^–1^ of the elution buffer (25 mm KPO_4_, pH 3). Detection of AsA and GSH occurred between 190 and 250 nm via a diode array (SPDM10AVP, Shimadzu, The Netherlands), set in tandem with a homemade amperometric detection system (glassy carbon working electrode, calomel reference electrode, reference potential 1000 mV). For PC analysis, samples were ground in 6·3 mm diethylenetriaminepentaacetic acid with 0·1 % (v/v) trifluoroacetic acid at 4 °C supplemented with 10 mm *N*-acetylcysteine as an internal standard. The samples were filtered and the thiols were derivatized with 25 mm monobromobimane (30 min, 45 °C). The thiols were separated at 37 °C on two tandemly arranged Nova-Pak C_18_ columns (6 nm, 4 μm, 3·9 × 150 mm, Waters, Milford, MA, USA), using a slightly concave gradient of 12–25 % (v/v) methanol (15 min) and then a linear gradient from 25 to 50 % (v/v) methanol (15–40 min). Fluorescence was monitored using a Waters 474 fluorescence detector. Quantification was based on the internal standard and GSH standards.

### RNA extraction, reverse transcription and gene expression

Samples were disrupted under frozen conditions using two stainless steel beads in a Retch Mixer Mill MM 2000 (Retsch, Germany). RNA was extracted using the RNAqueous Total RNA Isolation Kit (Ambion, Thermo Fisher Scientific, Waltham, MA, USA). The TURBO DNA-free kit (Ambion) and the High Capacity cDNA Reverse Transcription Kit (Ambion, random hexamer primers and 1 μg of RNA input) were used to remove genomic DNA and to carry out reverse transcription, respectively. A 10-fold dilution of the cDNA in 1/10 diluted TE-buffer (1 mm Tris–HCl, 0·1 mm EDTA, pH 8·0) was stored at –20 °C. Quantitative PCR (qPCR) was performed using Fast SYBR Green chemistry according to the manufacturer’s instructions on an ABI Prism 7500 Fast Real-Time PCR System (Applied Biosystems, Thermo Fisher Scientific, Waltham, MA, USA). Relative gene expression was calculated as 2^–ΔCq^ and was normalized with a normalization factor based on the expression of the following reference genes: *AT5G55840*, *AT2G28390* and *AT4G34270* for roots, and *AT2G28390*, *AT5G25760* and *AT4G34270* for leaves (Remans *et al*., 2008). Gene-specific primers (300 nm; Supplementary Data Table S1) were designed and optimized using Primer Express (Applied Biosystems). Supplementary Data Table S2 shows the reverse transcription quantitative (RTqPCR) parameters according to the Minimum Information for publication of RTqPCR Experiments (MIQE) guidelines (Bustin *et al*., 2009).

### Clustering of gene expression data

Hierarchical clustering analysis was performed (GenEx software, v6, MultiD Analyses AB, Sweden) to recognize potential sample-related patterns during Cd exposure in four *A. thaliana* genotypes (wild-type, *cad2-1*, *vtc1-1* and *cad2-1 vtc1-1*). The analysis was based on raw gene expression values and the ‘Average linkage’ algorithm, defining the distance between conditions as the average of distances between all pairs of individuals in all groups. Distances between the measures were calculated via the Euclidian Distance Measure. Heat maps were constructed to compare expression levels between different genes and samples.

### Statistical analysis

All data were analysed with general linear analysis of variance (ANOVA) models (Verbeke and Molenberghs, 2000). Normal distribution and homoscedasticity were tested using the Shapiro–Wilk and Bartlett test, respectively. Tukey post-hoc adjustment was used to correct for multiple comparison. Logarithmic transformations were applied when necessary to approximate normality; gene expression data were always log-transformed. All statistical analyses were performed using R (the R Foundation of Statistical Computing, version 2.15.1). Data are mean values ± standard error (s.e.) and significance was set at the 5 % level (*P* < 0·05).

## RESULTS

### Phenotypic characteristics of *Arabidopsis thaliana* genotypes: wild-type, glutathione-deficient, ascorbate-deficient and double mutant plants

**Fig. 1.**
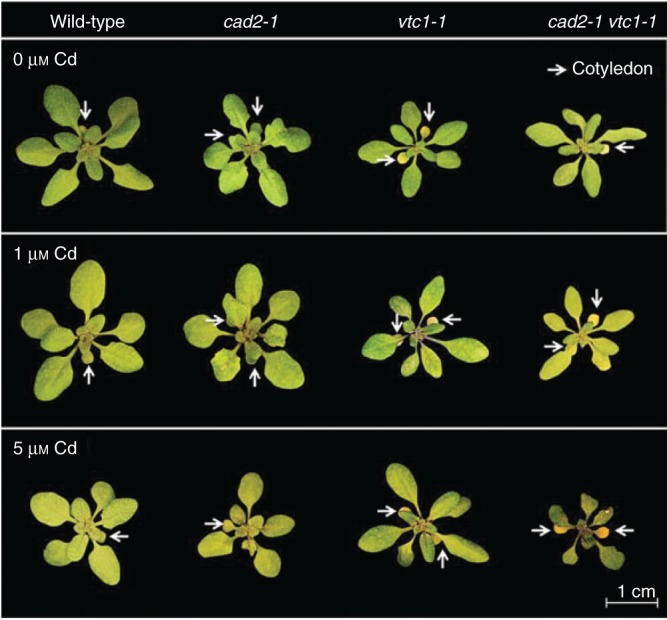
Representative pictures of the rosette appearance of four *Arabidopsis thaliana* genotypes (wild-type, *cad2-1*, *vtc1-1* and *cad2-1 vtc1-1*) exposed to 0, 1 or 5 μm CdSO_4_ for 72 h in hydroponics; *n* = 28. Cotyledons are indicated by white arrows.

After 3 weeks of growth under control conditions, wild-type and GSH-deficient plants (*cad2-1*) showed a similar phenotype, while both AsA-deficient mutants (*vtc1-1* and *cad2-1 vtc1-1*) differed from the wild-type plants. First, their rosettes showed a reduced growth and yellowing of the cotyledons (Fig. 1). Secondly, their root and leaf fresh weights were lower than in the wild type (Table 1). Although wild-type fresh weight only demonstrated an insignificant decreasing trend in response to Cd, a strong and significant decrease was observed for roots and leaves of *cad2-1* plants. The *vtc1-1* mutant, however, did not show any reduced growth after Cd exposure but its cotyledons turned more yellow (Fig. 1). The double mutant displayed a significantly reduced root fresh weight and its rosettes appeared small and with necrotic lesions in comparison with the control condition.

**Table1.** Biomass production (mg f. wt plant^–1^) of roots and leaves of four *Arabidopsis thaliana* genotypes (wild-type, *cad2-1*, *vtc1-1* and *cad2-1 vtc1-1*) exposed to 0, 1 or 5 μ*m* CdSO_4_ for 72 h in hydroponics

	F. wt (mg plant^–1^)	0 μm Cd	1 μm Cd	5 μm Cd
Leaves	Wild-type	95·75 ± 4·52^A^	73·80 ± 7·42^AB^	74·60 ± 5·35^AB^
	*cad2-1*	119·60 ± 14·89^A^	80·60 ± 8·49^AB^	41·10 ± 4·34^BC^
	*vtc1-1*	44·00 ± 1·78^BC^	43·00 ± 4·66^BC^	48·17 ± 10·91^BC^
	*cad2-1 vtc1-1*	32·75 ± 2·15^C^	24·48 ± 4·05^C^	28·21 ± 4·37^C^
Roots	Wild-type	70·00 ± 2·68^a^	42·70 ± 4·87^ab^	37·40 ± 3·45^ab^
	*cad2-1*	67·30 ± 8·98^a^	36·90 ± 6·29^ab^	12·57 ± 1·48^bc^
	*vtc1-1*	17·28 ± 1·89^bc^	8·26 ± 1·40^bc^	21·98 ± 7·61^bc^
	*cad2-1 vtc1-1*	9·14 ± 1·27^c^	7·24 ± 2·28^cd^	3·05 ± 0·57^d^

Statistical significance is expressed using lower (roots) and upper case letters (leaves); *n* = 5 (two-way ANOVA, *P* < 0·05).

### Genotype-specific accumulation of elements

**Fig. 2.**
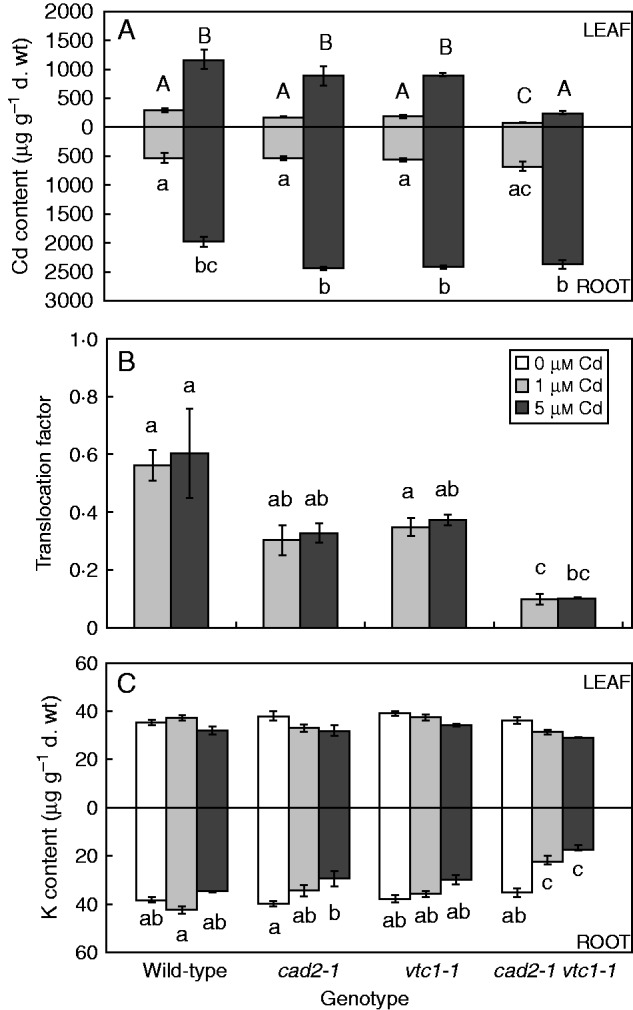
**(**A, C) Root and leaf element concentrations (μg g^–1^ d. wt) and (B) the corresponding translocation factor of Cd from roots to shoots of four *Arabidopsis thaliana* genotypes (wild-type, *cad2-1*, *vtc1-1* and *cad2-1 vtc1-1*) exposed to 0, 1 or 5 μm CdSO_4_ for 72 h in hydroponics. (A) Cadmium (Cd) content; and (C) potassium (K) content. Statistical significance is expressed using lower (roots and translocation factor) and upper case letters (leaves); *n* = 4 (two-way ANOVA, *P* < 0·05).

Exposure to Cd raised the Cd concentrations in a dose-dependent manner in both organs of all genotypes (Fig. 2A). However, the mutants showed increasing trends in root Cd levels and decreasing trends in the leaves. This resulted in lower Cd translocation factors in the mutants, though only significantly so in the double mutant (Fig. 2B). Concerning K, equal levels were found in all genotypes under control conditions (Fig. 2C). Exposure to Cd, however, resulted in lower K levels in roots of both GSH-deficient mutants (*cad2-1* and *cad2-1 vtc1-1*).

### Altered metabolite levels in arabidopsis genotypes

**Fig. 3.**
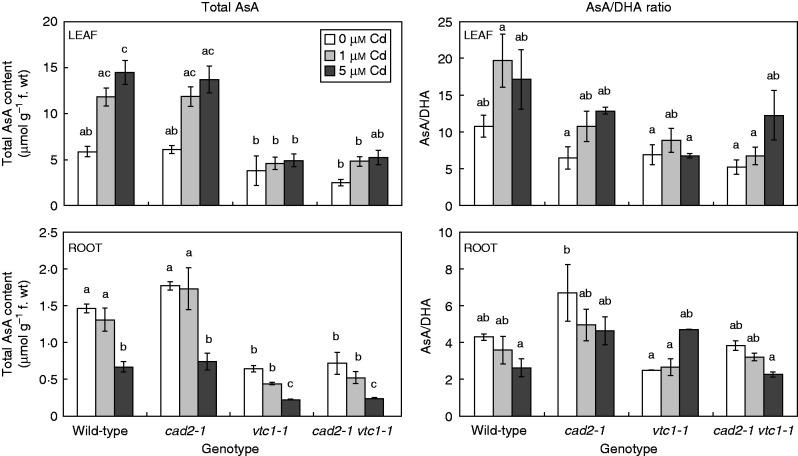
Concentration of total ascorbate (AsA) and the ratio between AsA and dehydroascorbate (DHA) in leaves and roots of four *Arabidopsis thaliana* genotypes (wild-type, *cad2-1*, *vtc1-1* and *cad2-1 vtc1-1*) exposed to 0, 1 or 5 μm CdSO_4_ for 72 h in hydroponics. Statistical significance is expressed using lower case letters; *n* = 4 (two-way ANOVA, *P* < 0·05).

**Fig. 4.**
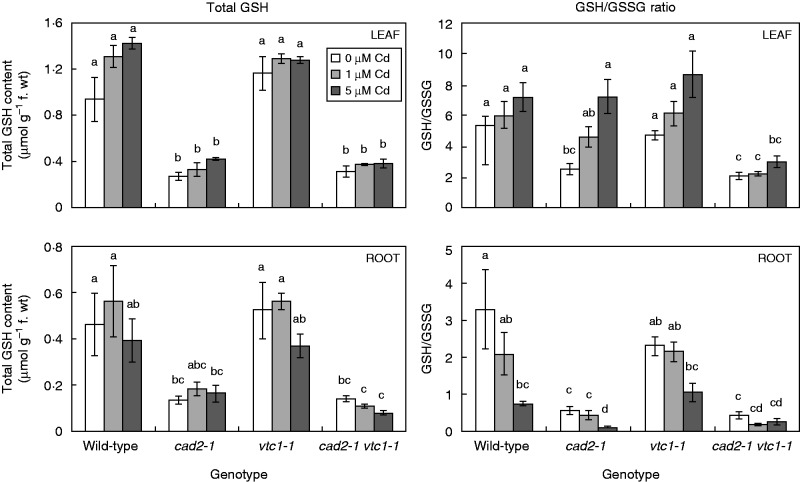
Concentration of total glutathione (GSH) and the ratio between GSH and glutathione disulphide (GSSG) in leaves and roots of four *Arabidopsis thaliana* genotypes (wild-type, *cad2-1*, *vtc1-1* and *cad2-1 vtc1-1*) exposed to 0, 1 or 5 μm CdSO_4_ for 72 h in hydroponics. Statistical significance is expressed using lower case letters; *n* = 4 (two-way ANOVA, *P* < 0·05).

Under control conditions, the mutant genotypes were verified: in comparison with wild-type plants, *vtc1-1* mutants contained 45–65 % AsA (Fig. 3), *cad2-1* mutants contained approx. 30 % GSH (Fig. 4), and *cad2-1 vtc1-1* mutants contained 40–50 % AsA and approx. 30 % GSH. Exposure to Cd resulted in a dose-dependent decrease in total AsA levels in the roots of all genotypes, while wild-type and *cad2-1* leaf AsA levels increased dose-dependently.

**Fig. 5.**
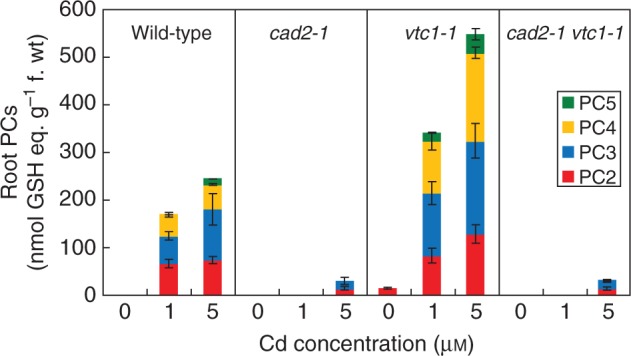
Profiling the phytochelatin (PC) content (see key) in four *Arabidopsis thaliana* genotypes (wild-type, *cad2-1, vtc1-1* and *cad2-1 vtc1-1*) in roots exposed to 0, 1 or 5 μm CdSO_4_ for 72 h in hydroponics (*n* = 4). Thiol content is expressed in GSH equivalents (nmol g^–1^ f. wt).

The GSH/GSSG ratio under control conditions was significantly lower in both GSH-deficient mutants in comparison with the other genotypes (Fig. 4). While Cd exposure resulted in a significantly lower GSH/GSSG ratio (i.e. a more oxidized GSH redox state) in roots of wild-type and *cad2-1* plants, both AsA-deficient mutants showed decreasing trends. In leaves, however, a generally more reduced GSH redox state was apparent, although only significantly so in leaves of *cad2-1* plants. Phytochelatins were absent under control conditions in roots, except in *vtc1-1* mutants, which contained a low concentration of PC2 (i.e. 14·75 nmol GSH equivalents g^–1^ f. wt) (Fig. 5). After exposure to Cd, a dose-dependent increase in root PCs was found in all genotypes. In the roots of *vtc1-1* plants, PC levels were approx. 2-fold higher than in wild-type plants under the same conditions. In both GSH-deficient genotypes, however, no PCs were detected after exposure to 1 μm Cd, and <15 % of wild-type PC levels were present after exposure to 5 μm Cd.

### Glutathione and ascorbate deficient plants possess a different gene expression profile

**Fig. 6.**
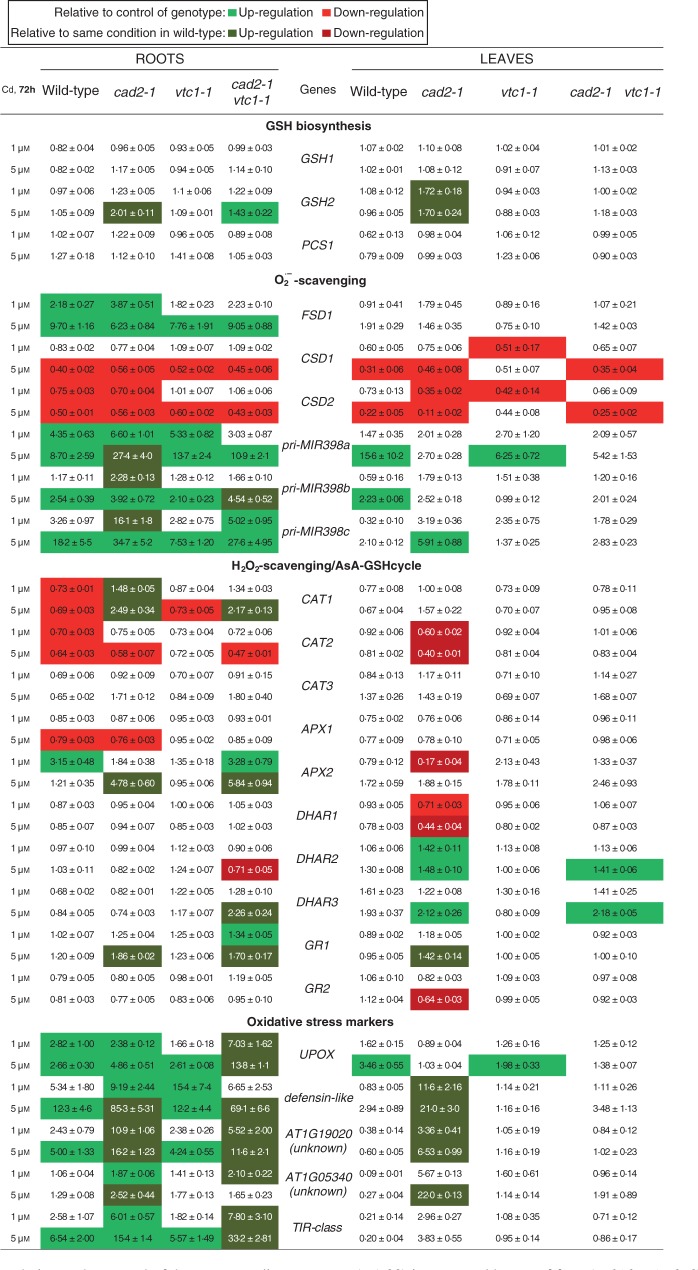
Gene expression patterns relative to the control of the corresponding genotype (=1·00) in roots and leaves of four *Arabidopsis thaliana* genotypes (wild-type, *cad2-1*, *vtc1-1* and *cad2-1 vtc1-1*). Plants were exposed to 1 or 5 μm CdSO_4_ for 72 h in hydroponics. Significant treatment effects relative to the control of the corresponding genotype (light green and light red, see key) and relative to the same condition in wild-type (dark green and dark red; two-way ANOVA; *P* < 0·05; *n* = 4). Genes: γ-glutamylcysteine synthetase (*GSH1*), glutathione synthetase (*GSH2*), phytochelatin synthase (*PCS1*), iron superoxide dismutase (*FSD1*), copper/zinc superoxide dismutase (*CSD1-2*), primary microRNA398 transcripts (*pri-MIR398a-c*), catalase (*CAT1-3*), ascorbate peroxidase (*APX1-2*), dehydroascorbate reductase (*DHAR1-3*), glutathione reductase (*GR*), Toll-Interleukin-1 class (*TIR*), upregulated by oxidative stress (*UPOX*).

**Fig. 7.**
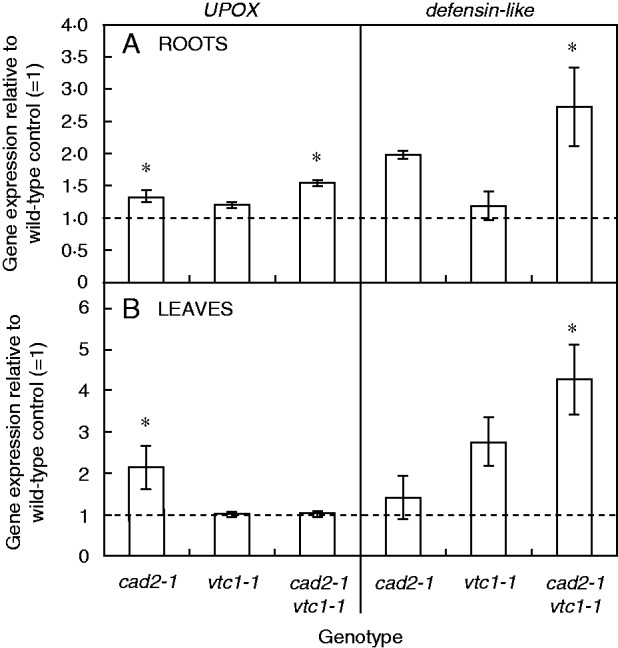
Gene expression of a gene upregulated by oxidative stress (*UPOX*) and a *defensin-like* gene in roots (A) and leaves (B) of three *Arabidopsis thaliana* mutants (*cad2-1*, *vtc1-1* and *cad2-1 vtc1-1*) under control conditions. Gene expression is expressed relative to the wild-type control (1: dashed line, *n* = 4, two-way ANOVA, significance level: *P < 0·05).

**Fig. 8.**
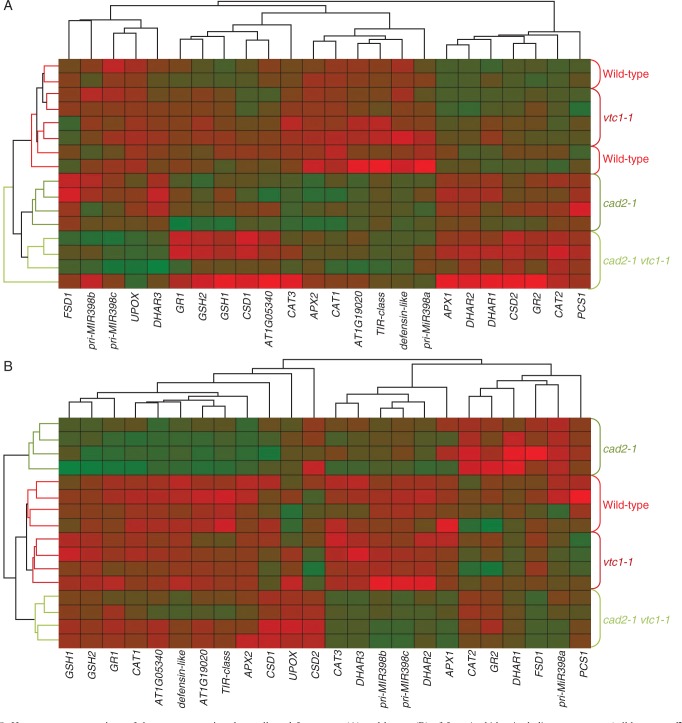
Heat map representations of the gene expression data collected from roots (A) and leaves (B) of four *Arabidopsis thaliana* genotypes (wild-type, *cad2-1*, *vtc1-1* and *cad2-1 vtc1-1*) exposed to 5 μM CdSO_4_ for 72 h in hydroponics (*n* = 4). Hierarchical clustering of genes is shown on the top and bottom; genotype clustering is visualized on the left and right. Green shaded boxes indicate high, and red shaded boxes low gene expression. Genes: γ-glutamylcysteine synthetase (*GSH1*), glutathione synthetase (*GSH2*), phytochelatin synthase (*PCS1*), iron superoxide dismutase (*FSD1*), copper/zinc superoxide dismutase (*CSD1-2*), primary microRNA398 transcripts (*pri-MIR398a-c*), catalase (*CAT1-3*), ascorbate peroxidase (*APX1-2*), dehydroascorbate reductase (*DHAR1-3*), glutathione reductase (*GR1-2*), Toll-Interleukin-1 class (*TIR*), upregulated by oxidative stress (*UPOX*).

In roots, only the GSH-deficient mutants significantly enhanced glutathione synthetase (*GSH2*) expression after exposure to 5 μm Cd (Fig. 6). However, all genotypes activated the SOD pathway in a dose-dependent manner, i.e. increasing FeSOD (*FSD1*) and microRNA398 [primary microRNA398 transcripts (*pri-MIR398a-c*)] expression, resulting in decreasing CuZnSOD (*CSD1/2*) transcript levels. Both *CAT1* and *CAT2* were downregulated after Cd exposure, except in the GSH-deficient mutants, where *CAT1* was significantly upregulated in a dose-dependent manner. Although *APX1* was downregulated after Cd exposure, *APX2* was strongly induced in wild-type plants exposed to 1 μm Cd and in a dose-dependent manner in the GSH-deficient mutants, but not in the roots of *vtc1-1* plants. No significant Cd-induced changes were detected in *DHAR* expression except in the double mutant, which showed significantly decreased *DHAR2* and increased *DHAR3* transcript levels after exposure to 5 μm Cd. The roots of *cad2-1* and double mutant plants showed an increase in *GR1* transcript levels that was the highest after 5 μm Cd exposure. Finally, Cd exposure induced oxidative stress marker genes in roots of all genotypes in a dose-dependent manner (Fig. 6). On the one hand, in the roots of wild-type and *vtc1-1* plants, three out of five investigated genes were upregulated (>5-fold) to the same extent. On the other hand, both GSH-deficient mutants enhanced all genes and often to a significantly greater extent than in roots of wild-type plants. Furthermore, these mutants possessed elevated transcript levels of only two oxidative stress marker genes (i.e. *UPOX* and the *defensin-like* gene) in both organs under control conditions (Fig. 7). When representing the gene expression data together in a heat map, hierarchical agglomerate clustering revealed two clusters in roots of plants exposed to 5 μm Cd (Fig. 8). One cluster contained wild-type and *vtc1-1* plants showing mostly downregulated expression levels, and the second cluster included both GSH-deficient mutants of which the roots of *cad2-1* plants displayed more upregulated genes, while the double mutant showed a combination of both colours evenly distributed (Fig. 8A).

**Fig. 9.**
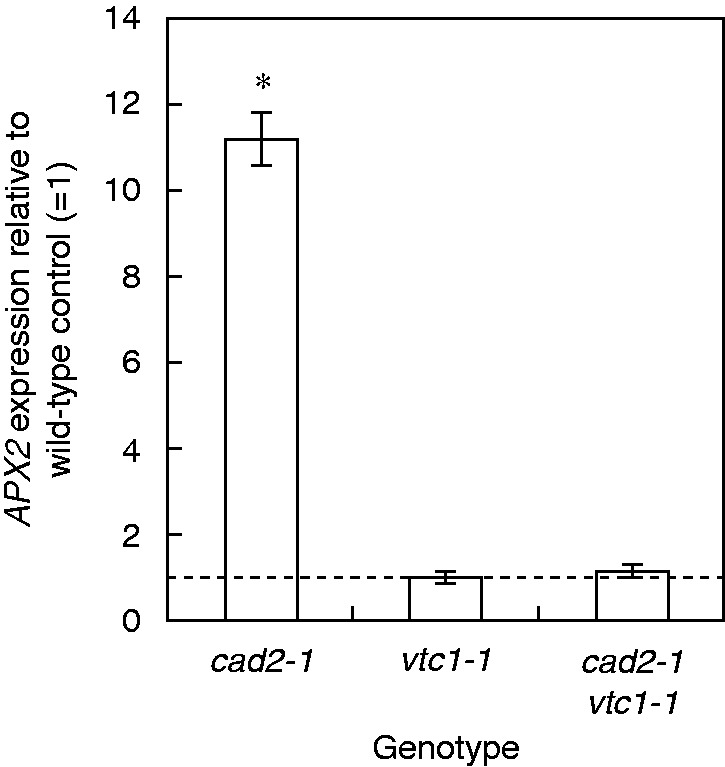
Gene expression of ascorbate peroxidase 2 (*APX2*) in leaves of three *Arabidopsis thaliana* mutants (*cad2-1*, *vtc1-1* and *cad2-1 vtc1-1*) under control conditions. Gene expression is expressed relative to the wild-type control (1: dashed line, *n* = 4, two-way ANOVA, significance level: *P < 0·05).

In leaves, only the *cad2-1* mutant increased *GSH2* expression after Cd exposure (Fig. 6). The SOD pathway was activated but to a smaller extent than in roots: *CSD1/2* transcripts were decreased and *pri-MIR398a-c* was significantly up-regulated in some conditions, but no significant induction of *FSD1* was present. In the leaves, *cad2-1* plants showed a significant Cd-induced downregulation of *DHAR1*, but a significant upregulation of *DHAR2-3*. Also the double mutant showed increased *DHAR2-3* transcript levels after Cd exposure. In addition, the leaves of *cad2-1* plants exhibited an increased *GR1* and a decreased *GR2* expression after exposure to the highest Cd concentration. In leaves of both wild-type and *vtc1-1* plants exposed to 5 μm Cd, the expression level of one oxidative stress marker gene was significantly induced by Cd exposure but only to a minor extent. In the double mutant, no significant Cd-induced changes were detected, while the leaves of *cad2-1* plants showed >5-fold increased expression levels of three oxidative stress marker genes. Finally, an interesting genotype effect was detected in the leaves of *cad2-1* plants under control conditions: *APX2* transcripts were >10-fold higher in comparison with wild-type plants (Fig. 9). The heat map for leaves exposed to 5 μm Cd demonstrates that the *cad2-1* mutant did not cluster with the other genotypes (Fig. 8B).

## DISCUSSION

**Fig. 10.**
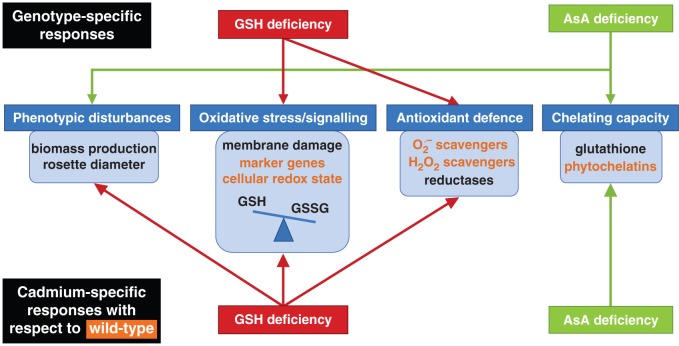
Schematic overview of both genotype-specific responses under control conditions (upper part) and cadmium (Cd)-specific responses (lower part). Wild-type responses under Cd stress are indicated in orange. Red and green arrows represent additional responses with respect to the wild-type responses in mutants deficient in glutathione (GSH) and ascorbate (AsA), respectively. Abbreviations: GSSG, glutathione disulphide; O_2_·^–^, superoxide; H_2_O_2_, hydrogen peroxide.

Upon exposure to toxic Cd concentrations, plants activate their defence strategies related to both the reduction of the free metal content in the cell and the regulation of antioxidative defence mechanisms. Glutathione plays a key role as chelator due to the high affinity of Cd for its thiol group and as a precursor of PCs. Antioxidative defence mechanisms are largely based on the use and recycling of both GSH and AsA, complemented by SOD and CAT. In addition to their function as primary antioxidants, GSH and AsA act as substrates for the regeneration of other essential antioxidants. Together, these functions influence the concentration and cellular redox state, i.e. reduction/oxidation ratio, of free reduced metabolites (Jozefczak *et al*., 2012; Bielen *et al*., 2013). Therefore, both parameters concerning GSH and AsA were taken into consideration in *A. thaliana* plants exposed to environmentally realistic Cd concentrations (1 and 5 μm) during 72 h (for a schematic overview, see Fig. 10) (Krznaric *et al*., 2009). In general, roots of Cd-exposed wild-type plants were in a more stressed condition than leaves, evidenced by a more oxidized cellular redox state (Figs 3 and 4) and enhanced oxidative stress marker gene induction (Fig. 6). The absence of K leakage from roots (Fig. 2C), which is associated with metal-induced membrane damage (Demidchik *et al*., 2010), confirms that the roots were able to cope with the elevated metal concentrations (Keunen *et al*., 2011*b*). In agreement with earlier findings, they responded via increasing both their chelating capacity via PC production (Fig. 5) and their antioxidative capacity via upregulation of ROS-scavenging genes (Fig. 6) (DalCorso *et al*., 2008; Jozefczak *et al*., 2014). Cadmium retention and detoxification in roots contributed to lower Cd levels in the leaves (Fig. 2A). Together with high levels of GSH and AsA and highly reducing redox potentials, a well-balanced cellular redox state and an optimal function of the AsA–GSH cycle were maintained in the leaves of wild-type plants (Figs 3 and 4). Several studies reported increased enzyme activities concerning this cycle, highlighting its relevance in Cd defence (Paradiso *et al*., 2008; Drazkiewicz *et al*., 2010).

### Cadmium-sensitive glutathione-deficient mutants perceive permanent oxidative stress

Glutathione and AsA are abundant and multifunctional metabolites in plants. In this study, the consequences of deficiency in any of these two metabolites were investigated by including mutants (*cad2-1*, *vtc1-1* and *cad2-1 vtc1-1*) which demonstrated multiple genotype-specific effects (for a schematic overview, see Fig. 10). Although the GSH-deficient *cad2-1* mutant did not exhibit any growth phenotype, both GSH-deficient mutants suffered from oxidative stress under control conditions [i.e. a more oxidized GSH pool (Fig. 4) and elevated expression of oxidative stress marker genes (Fig. 6)]. In addition, several studies suggested that changes in the GSH redox state regulate multiple cellular processes at the gene expression and protein level, as indicated by the elevated *APX2* expression in the leaves of *cad2-1* plants (Fig. 9) (Cobbett *et al*., 1998; Mou *et al*., 2003; Ball *et al*., 2004; Parisy *et al*., 2007; Jozefczak *et al*., 2012). This confirms that the GSH redox state is an important indicator of a cell’s biological status, thus reflecting cellular toxicity (Schäfer and Buettner, 2001; Foyer and Noctor, 2011; Jozefczak *et al*., 2012). Recent *in vivo* studies, using a redox-sensitive fluorescent probe in GSH-deficient mutants, associated increased susceptibility to pathogens with a more oxidized GSH redox potential (Parisy *et al*., 2007; Dubreuil-Maurizi *et al*., 2011). We suggest that both decreased GSH levels and a more oxidized GSH pool contribute to increased sensitivity to Cd.

Under Cd exposure, GSH-deficient mutants showed a lower Cd translocation to the shoots (Fig. 2B), which is in agreement with a recent study (Sobrino-Plata *et al*., 2014). Nevertheless, this resulted in elevated toxicity responses and oxidative stress upon Cd exposure. Similar to wild-type plants, the roots of *cad2-1* mutants were generally more stressed than the leaves. The more oxidized environment in the mutant under control conditions contributed to the activation of alternative pathways using both O_2_·^–^ and H_2_O_2_ scavengers in order to adapt to the increasing Cd levels, while wild-type plants with a higher level of total GSH and a higher GSH/GSSG ratio did not. These findings support the fact that changes in the cellular redox state act on the regulation of gene expression through oxidation or reduction of transcription factors (Apel and Hirt 2004; Mittler *et al*., 2004). In general, a decreased capacity to chelate Cd ions, in combination with a more oxidized GSH pool, was associated with the activation of antioxidative defence systems upon Cd exposure, which were not required under wild-type conditions. Together, these data confirm the essential role of the GSH redox state and concentration in elevated sensitivity to Cd exposure in GSH-deficient mutants (Howden *et al*., 1995; Jozefczak *et al*., 2012).

### Elevated thiol levels in ascorbate-deficient plants protect them against cadmium stress

The importance of AsA and its precursors in cell proliferation, elongation and cell wall synthesis (Veljovic-Jovanovic *et al*., 2001; Bielen *et al*., 2013) was confirmed by the slow-growth phenotypes of the AsA-deficient mutants (Fig. 1; Table 1). Despite decreased AsA levels, *vtc1-1* plants showed a reduced cellular redox state similar to wild-type plants under control conditions (Figs 3 and 4), which was confirmed by the lack of induction of oxidative stress marker genes (Fig. 7). An interesting difference from wild-type plants, however, was the elevated thiol concentration detected in unexposed *vtc1-1* plants (Fig. 5). Increased basal GSH levels have been observed before in these mutants (Veljovic-Jovanovic *et al*., 2001; Pavet *et al*., 2005). In addition, AsA deficiency was previously suggested to provide a primed state that decreases pathogen susceptibility in *A. thaliana*. This priming was related to elevated levels of phytohormones such as abscisic acid and gibberellic acid, i.e. components involved in developmental and defence signalling (Pastori *et al*., 2003; Barth *et al*., 2004; Pavet *et al*., 2005; Mukherjee *et al*., 2010; Brosché and Kangasjarvi, 2012). We hypothesize that AsA deficiency also prepares *A. thaliana* plants for abiotic stresses such as Cd exposure by stimulating their thiol production.

In general, *vtc1-1* mutants showed a similar transcript profile to wild-type plants concerning both the antioxidant and oxidative stress marker genes (Fig. 6). Additionally, in comparison with wild-type plants, these mutants were more able to maintain a stable and reducing environment concerning the AsA and GSH redox state (Figs 3 and 4), indicative of an active recycling of both metabolites (Conklin and Barth, 2004). Together, these data support the hypothesis that AsA deficiency renders arabidopsis mutants less sensitive to Cd. In addition to the elevated thiol levels found under control conditions, PC levels were approximately twice as high in the roots of *vtc1-1* than in wild-type plants upon Cd exposure (Fig. 5). As one of the major defence mechanisms in Cd-exposed plants is fast chelation and sequestration by thiols (Zhang *et al*., 2013; Jozefczak *et al*., 2014), the enhanced chelating capacity in *vtc1-1* plants is suggested to contribute to a less Cd-sensitive phenotype. These findings are consistent with the study of Brosché and Kangasjarvi (2012) who summarized that low AsA conditions led to opposite phenotypes compared with low GSH conditions after multiple stresses, including ozone treatment and pathogen infection.

### Conclusions

Our observations indicate that GSH and AsA deficiency differentially alter plant GSH homeostasis, resulting in opposite Cd sensitivities relative to wild-type plants (for a schematic overview, see Fig. 10). Under control conditions (upper part of Fig. 10), GSH deficiency resulted in elevated oxidative stress and expression of the antioxidant APX2. Deficiency in AsA, however, resulted in phenotypic disturbances and elevated basal thiol levels. Upon Cd exposure (lower part of Fig. 10), GSH-deficient mutants were hampered in chelation. They experienced phenotypic disturbances and even more oxidative stress, and therefore activated multiple alternative pathways such as SOD, CAT and APx, indicating a higher Cd sensitivity. Ascorbate deficiency, however, was associated with enhanced PC synthesis after Cd exposure, in comparison with wild-type plants, which contributed to decreased sensitivity towards Cd.

## SUPPLEMENTARY DATA

Supplementary data are available online at www.aob.oxfordjournals.org and consist of the following. Table S1: list of primers used in the reverse transcription–quantitative PCR. Table S2: reverse transcription–quantitative PCR parameters.

Supplementary Data
