# Supplementary material for: Both the concentration and redox state of glutathione and ascorbate influence the sensitivity of arabidopsis to cadmium
Source: Ann Bot. 2015 Jun 12;116(4):601–12. doi: 10.1093/aob/mcv075 (PMC4577996; doi:10.1093/aob/mcv075)
Supplement: Supplementary Data [file supp_116_4_601__index.html]

Both the concentration and redox state of glutathione and ascorbate influence the sensitivity of arabidopsis to cadmium — Supplementary Data 

# Both the concentration and redox state of glutathione and ascorbate influence the sensitivity of arabidopsis to cadmium

## Supplementary Data

files

- Supplementary Data - pdf file
