# Supplementary material for: Both the concentration and redox state of glutathione and ascorbate influence the sensitivity of arabidopsis to cadmium
Source: Ann Bot. 2015 Jun 12;116(4):601–12. doi: 10.1093/aob/mcv075 (PMC4577996; doi:10.1093/aob/mcv075)
Supplement: Supplementary Data [file supp_mcv075_aob-15046-s01.pdf]

SUPPLEMENTARY DATA

Table S1. List of primers used in the reverse transcription quantitative PCR. Exon-Exon-junction (E-E-jn), Intron-Exon-junction (In-E-jn).

Gene	Locus	Annotation	Primer sequences	Exon location	Amplicon size	Primer efficiency
APX1	AT1G07890	Ascorbate peroxidase1	FW: TGCCACAAGGATAGGTCTGG<br>REV: CCTTCCTTCTCTCCGCTCAA	Exon 5	101 bp	96.3 %
APX2	AT3G09640	Ascorbate peroxidase2	FW: TTGCTGTTGAGATCACTGGAGGA<br>REV: TGAGGCAGACGACCTTCAGG	Exon 3	91 bp	90.9 %
AT1G19020	AT1G19020	Unknown oxidative stress marker	FW: GAAAATGGGACAAGGGTTAGACAAA<br>REV: CCCAACGAAAACCAATAGCAGA	Exon 1	92 bp	95.7 %
AT1G05340	AT1G05340	Unknown oxidative stress marker	FW: TCGGTAGCTCAGGGTAAAGTGG<br>REV: CCAGGGCACAACAGCAACA	Exon 2	91 bp	99.1 %
CAT1	AT1G20630	Catalase1	FW: AAGTGCTTCATCGGGAAGGA<br>REV: CTTCAACAAAACGCTTCACGA	E5-E6-jn	103 bp	97.6 %
CAT2	AT4G35090	Catalase2	FW: AACTCCTCCATGACCGTTGGA<br>REV: TCCGTTCCCTGTCGAAATTG	Exon 2	76 bp	98.3 %
CAT3	AT1G20620	Catalase3	FW: TCTCCAACAACATCTCTTCCCTCA<br>REV: GTGAAATTAGCAACCTTCTCGATCA	Exon 2	91 bp	95.6 %
CSD1	AT1G08830	CuZn superoxide dismutase1	FW: TCCATGCAGACCCTGATGAC<br>REV: CCTGGAGACCAATGATGCC	Exon 5	102 bp	93.8 %
CSD2	AT2G28190	CuZn superoxide dismutase2	FW: GAGCCTTTGTGGTTCACGAG<br>REV: CACACCACATGCCAATCTCC	Exon 6	101 bp	93.9 %
defensin-like	AT2G43510	Defensin-like	FW: ATGGCAAAGGCTATCGTTTCC<br>REV: CGTTACCTTGCGCTTCTATCTCC	Exon 1	91 bp	96.8 %
DHAR1	AT5G16710	Dehydroascorbate reductase1	FW: CCAGATTCACTTCCTTTCGTCAA<br>REV: TTACATCCTCTGTTTCCGCCC	Exon 6	91 bp	94.0 %
DHAR2	AT1G75270	Dehydroascorbate reductase2	FW: ATCAGATGGGTCTTGTAGGGAAGC<br>REV: GTGCTCCTGATGTTCTCGGC	Exon 2	91 bp	95.1 %
DHAR3	AT1G19550	Dehydroascorbate reductase3	FW: AACTCTTTCCCCGGCGATAA<br>REV: CTGAATTTGCCTCTGTTGGCTC	Exon 1	92 bp	95.2 %
FSD1	AT4G25100	Fe superoxide dismutase1	FW: CTCCCAATGCTGTGAATCCC<br>REV: TGGTCTTCGGTTCTGGAAGTC	Exon 4	101 bp	88.8 %
GR1	AT3G24170	Glutathione reductase1	FW: CTCAAGTGTGGAGCAACCAAAG<br>REV: ATGCGTCTGGTCACACTGC	Exon 15	101 bp	94.79%
GR2	AT3G54660	Glutathione reductase2	FW: GCCCAGATGGATGGAACAGAT<br>REV: TAGGGTTGGAGAATGTTGGCG	Exon 5	91 bp	96.4 %

Gene	Locus	Annotation	Primer sequences	Exon location	Amplicon size	Primer efficiency
GSH1	AT4G23100	γ-Glutamylcysteine synthetase	FW: CCCTGGTGAACTGCCTTCA<br>REV: CATCAGCACCTCTCATCTCCA	Exon 5	101 bp	98.6 %
GSH2	AT5G27380	Glutathione synthetase	FW: GGACTCGTCGTTGGTGACAA<br>REV: TCTGGGAATGCAGTTGGTAGC	Exon 11	101 bp	92.6 %
pri-MIR398a	AT2G03445	Primary microRNA398a	FW: AGAAGAAGAGAAGAACAACAGGAGGTG<br>REV: ATTAGTAAGGTGAAAAAATGG	In1-E1-jn	156 bp	96.5 %
pri-MIR398b	AT5G14545	Primary microRNA398b	FW: AGTAATCAACGGCTGTAATGACGCTAC<br>REV: TGACCTGAGAACACATGAAAACGAGAG	Exon 1	67 bp	85.3 %
pri-MIR398c	AT5G14545	Primary microRNA398c	FW: TCGAAACTCAAACTGTAACAGTCC<br>REV: ATTTGGTAAATGAATAGAAGCCACGGGCCACG	Exon 1	241 bp	104.2 %
PCS1	AT5G44070	Phytochelatin synthase1	FW: TGCCAAGGAGCTGAAATCTT<br>REV: ACCGTGCCTTCAGAGTCATC	Exon 2	91 bp	95.1 %
PPR	AT5G55840	Pentatricopeptide repeat protein	FW: AAGACAGTGAAGGTGCAACCTTACT<br>REV: AGTTTTTGAGTTGTATTTGTCAGAGAAAG	Intron 2	59 bp	81.5 %
SAND	AT2G28390	SAND family	FW: AACTCTATGCAGCATTTGATCCACT<br>REV: TGATTGCATATCTTTATCGCCATC	Exon 13	61 bp	107.8 %
Tip41	AT4G34270	Tip41-like protein	FW: GTGAAAACTGTTGGAGAGAAGCAA<br>REV: TCAACTGGATACCCTTTCGCA	E1-E2-jn	61 bp	91.2 %
TIR-class	AT1G57630	Toll-interleukin-1 class	FW: ACTCAAACAGGCGATCAAAGGA<br>REV: CACCAATTCGTCAAGACAACACC	Exon 1	91 bp	97.3 %
UBC	AT5G25760	Ubiquitin conjugating enzyme	FW: CTGCGACTCAGGGAATCTTCTAA<br>REV: TTGTGCCATTGAATTGAACCC	E3-E4-jn	61 bp	98.7 %
UPOX	AT2G21640	Up-regulated by oxidative stress	FW: GACTTGTTTCAAAAACACCATGGAC<br>REV: CACTTCCTTAGCCTCAATTTGCTTC	Exon 1	91 bp	95.9 %

Table S2. Reverse transcription quantitative PCR parameters according to the Minimum Information for publication of Quantitative real-time PCR Experiments (MIQE) précis checklist derived from Bustin *et al*., 2009.

**Sample/Template**	
Source	*Arabidopsis thaliana* roots or shoots in a hydroponic culture
Method of preservation	harvest in liquid N_2 then stored at -80°C
Storage time	three weeks
Handling	frozen
Extraction method	columns: RNAqueous Total RNA Isolation Kit* (Ambion, Lennik, Belgium)
RNA: DNA-free	TURBO DNA-free Kit* (Ambion, Lennik, Belgium)
	intron-spanning primers and verification of single peak on melt curves
Concentration	NanoDrop®: ND-1000 Spectrofotometer (Isogen Life Science, IJsselstein, the Netherlands)
RNA: integrity	Microfluidics: Bioanalyzer* (Agilent Technologies, Waldbronn, Duitsland)
	for a representative subset of the samples
**Assay optimisation/validation**	
Accession number	see Table S.1
Amplicon details	exon location & amplicon size: see Table S.1
Primer sequence	see Table S.1
In silico	Primer-BLAST
Empirical	primer concentration (300 nM), annealing temperature (60°C)
Priming conditions	random hexamer priming
PCR efficiency	dilution curves (slope, deviation, see Table S.1)
Linear dynamic range	samples are within the efficiency curve
**RT/PCR**	
Protocols	TURBO DNA-free Kit* (Ambion, Lennik, Belgium)
	Fast SYBR Green* (Applied Biosystems, Paisley, UK)
	see Materials and Methods
Reagents	see Materials and Methods
NTC	C_q & melt curves
**Data analysis**	
Specialist software	7500 Fast System Sequence Detection Software, version 1.4
	(Applied Biosystems, Lennik, Belgium, 2001-2006)
Statistical justification	4 biological replicates, two-way ANOVA
	and Tukey post-hoc adjustment for multiple comparison
Normalisaton	3 reference genes selected using geNorm, version 3.5
	(Center for Medical Genetics, Ghent, Belgium, 2001-2007)

*All procedures were performed according to the manufacturer's protocol.
